# Portion Size of Energy-Dense Foods among French and UK Adults by BMI Status

**DOI:** 10.3390/nu11010012

**Published:** 2018-12-20

**Authors:** Holly L. Rippin, Jayne Hutchinson, Jo Jewell, Joao J. Breda, Janet E. Cade

**Affiliations:** 1 Nutritional Epidemiology Group (NEG), School of Food Science and Nutrition, University of Leeds, Leeds LS2 9JT, UK; J.Hutchinson1@leeds.ac.uk (J.H.); J.E.Cade@leeds.ac.uk (J.E.C.); 2 Division of Noncommunicable Diseases and Promoting Health through the Life-Course, World Health Organization Regional Office for Europe, UN City, Marmorvej 51, DK-2100 Copenhagen, Denmark; jewellj@who.int (J.J.); rodriguesdasilvabred@who.int (J.J.B.)

**Keywords:** food portion size, BMI status, energy-dense foods, national diet surveys, WHO European region, nutritional epidemiology

## Abstract

Evidence links consumed food portion size (FPS) and excess weight via increased energy intake. Policies to regulate on-pack serving sizes may be needed; determining consumed FPS of popular energy-dense foods for normal weight and overweight or obese (OWOB) adults, as reported here, may provide evidence to assist this. Data were analysed from national cross-sectional surveys, the French Étude Individuelle Nationale des Consommations Alimentaires2 2005–2007 (*n* = 2117), and UK National Diet and Nutrition Survey 2008–2014 (*n* = 3413). The impact of body mass index (BMI) on FPS is also investigated, adjusting for age, sex and under-reporting. Effects of under-reporting on relationships between FPS and BMI; and BMI on consumption frequency (UK only) were explored. OWOB reported larger FPS than normal-weight individuals in many, but not all food subgroups; however, there were only two significant FPS differences. In adjusted analyses, French individuals consumed 1.0 g (99% CI 0.01–2.1 *p* = 0.01) greater FPS in cakes for 1 point difference in BMI. ‘Other cakes’ and ‘dark chocolate’ were also significantly positively associated with BMI. High-fat bar snacks, but no UK main food groups, were positively associated with BMI. There was limited evidence of links between FPS and BMI in UK and French national cross-sectional data, possibly due to data limitations such as under-reporting. Future work should explore this and relationships between consumed FPS and on-pack suggested serving sizes to provide evidence to assist obesity-prevention policies.

## 1. Introduction

Europe is the World Health Organization (WHO) region most severely affected by non-communicable diseases (NCDs), which, alongside related conditions including overweight and obesity, have significant and growing economic and social costs. It is accepted that obesity is one of the biggest health problems facing the European population; it accounts for 2–8% of WHO European health costs [1] and is estimated to cause 320,000 deaths annually in Western Europe alone [2]. In 87% (46) of WHO European countries, over half of adults are overweight or obese (OWOB); in France 47% adults and in the UK 67% men and 58% women are OWOB [3,4]. However, despite OWOB rates being lower in France, prevalence has stabilised in the UK and increased in France in the last decade [5].

Evidence indicates an indirect link between food portion size (FPS) and excess weight via increased energy intake, and suggests that limiting FPS contributes to reduced energy intake and, therefore, reduced weight gain [6,7,8]. Both Ledikwe, et al. [6] and Bhupathiraju and Hu [9] link large FPS, particularly of energy-dense foods, with rising adult obesity levels in America via elevated energy intakes. European studies of adults are lacking, although Albar et al. [10] found positive associations between body mass index (BMI) and FPS of biscuits and cakes in UK adolescents, and Lioret et al. [11] found similar positive associations in croissants and sweetened pastries in French children. The evidence supports the WHO stance that ‘energy dense, micronutrient poor foods’ high in energy, saturated fats, trans fats, sugar and salt should be limited for a healthy diet [2]. There is some recognition of FPS as a policy tool to deliver this, forming part of the UK’s ongoing calorie reduction drive. The Public Health Responsibility Deal calorie reduction pledge included FPS reduction in its suite of options and Public Health England highlight it in their recent calorie reduction plans [12,13]. However, more policy focus is needed; based on a systematic review Marteau et al. argue that policy does not adequately reflect the importance of consumed portion size in excess weight and that effective interventions could potentially reduce energy intake in UK adults by 12–16% [14,15].

The positive association between FPS and food intake is known as the ‘portion size effect’ [16]. In their meta-analysis of experimental studies, Zlatevska et al. [17] found that although the association was not linear or uniform across all population groups, overall energy intake increased by 35% when FPS doubled. Kelly et al. [18] also found that adults consumed higher portions of pre-packed foods when food was presented as larger portion sizes, although this was a pilot study and potentially underpowered.

Evidence suggests that UK commercially available FPS have increased in some energy-dense food categories in the 20 years since government-based FPS guidelines [19] were released [20]. Although some varieties of traditional packet items like biscuits and crisps changed little during this time, other food categories have increased, including fast food and confectionary [21,22,23]. Although there is little corresponding literature on changing French FPS over the same 20-year period, the rise in French OWOB levels over the past decade [5] suggests that increased FPS may be a possibility.

National diet survey (NDS) methodologies are developed primarily to assess the nutritional status of a population [24], so are a suitable means of investigating overweight and obesity across Europe. In addition to both being developed Western European countries with similarities in food consumption, the French and UK NDS use similar dietary assessment methodology and their data were available for analysis. This makes them a good comparative case, and provides some, if limited, insight into continental Europe. In their review of recent developments regarding portion size mechanisms, interventions and the portion size effect, Steenhuis and Poelman [20] conclude that there is extensive evidence for the portion size effect on energy intake, but portion size policies and their acceptance by the public is an understudied area. Therefore, more information on consumed FPS, such as reporting by BMI status, and the effects of under-reporting, could add to the body of knowledge needed to fill this gap. These are complex issues that require detailed understanding of food groups and culture; here we present a comparison of two countries—further work could explore others.

Using their respective NDS [25,26,27], this paper will identify commonly consumed energy, fat and sugar-dense snack-type foods in French and UK adults, and determine their consumed FPS. Providing estimated consumed FPS for these energy-dense foods could be useful for policymakers regarding decisions around setting on-pack serving sizes, which are not currently regulated or standardised. Consumed FPS of normal weight, feasible reporters may be the most suitable reference when setting on-pack serving sizes, and focusing on frequently consumed energy-dense, high-fat and sugar snack foods may have the greatest impact on population overweight and obesity. The aim of this research is to examine how FPS might vary with BMI, and explore how FPS may be affected by under-reporting. FPS of the two countries will be reported, and associations between consumption frequency and BMI considered.

## 2. Materials and Methods

Two government-funded NDS were obtained and analysed for consumed FPS for selected food groups in relation to BMI in adults aged 19–64 years. Consumed FPS was defined as the total weight (in grams) of a particular food consumed in one eating occasion.

### 2.1. National Diet Survey (NDS) Data

Data collected for the French Étude Individuelle Nationale des Consommations Alimentaires 2 (INCA2) from 2005–2007 were used in the analyses [25]. The study population was taken from randomly selected primary geographical, then household units, using the 1999 Institut National de la Statistique et des Etudes Economiques (INSEE) national census, and weighted by sex, age, profession, head of household social category, season, region, size of household and urban area. Food data were collected from individuals aged 3–79 years via a consecutive 7-day diary, and FPS given either by grams per unit, household measures or using a photograph manual. Foods were coded using the INCA2 nomenclature, compatible with the CIQUAL food composition database [28]. Demographic information was gathered by a computer-assisted personal interview (CAPI) delivered by a trained professional, who also weighed and measured participants to calculate BMI. Nutrient intakes were derived from dietary data using the CIQUAL food composition database [25].

UK data were analysed from the National Diet and Nutrition Survey (NDNS) Rolling Programme (RP) years 1–4 and 5–6 (2008–2012 and 2012–2014) [26,27]. The two datasets were appended and sample weightings adjusting for unequal selection probability and non-response were reassigned. Existing weightings for the Y1–4 and Y5–6 datasets were rescaled to ensure the two datasets were in the correct proportion to produce approximately nationally representative results; separate weightings for adults aged 19–64 years were created using this process [29] (see Supplementary Material S1). Similar to the French INCA2, multi-stage random probability sampling from UK postcode address files with postal sectors as the primary sampling units was used to select individuals for the UK NDNS. Food data were collected from individuals aged 1.5–100 y via a 4-day consecutive food diary, and portion sizes estimated using household measures, photographs and food packaging labels [30]. Demographic information was also collected by a trained interviewer via a CAPI and measured height and weight gathered for BMI. Nutrient intakes were derived from dietary data using the McCance and Widdowson’s composition of foods integrated dataset [26,27].

Data from 2117 French and 3413 UK adults aged 19–64 years were used for the overall analyses. Underweight adults with a BMI <18.5 were excluded from analyses; this constituted 98 individuals in the French INCA2 and 46 in the UK NDNS. In addition, 25 adults with missing BMI values were excluded from analyses in the INCA2 and 203 adults with missing BMI or infeasible BMR:energy intake values excluded from the NDNS.

### 2.2. Preparation of Variables

The NDNS 2008–2014 dataset was used to identify popular energy, fat and sugar-dense foods in the UK. The main NDNS food groups were listed by total number of eating occasions for adults aged 19–64 years and the tertile with the lowest number of eating occasions were excluded. The average energy, fat and total sugar density was then determined for each remaining food groups and those with above average density in all three nutrients were selected for analysis—these were biscuits, buns, cakes, pastries and fruit pies (hereby referred to as ‘Cakes’) and chocolate confectionary (hereby referred to as ‘Chocolate’). Although crisps and savoury snacks (hereby referred to as ‘Crisps’) did not have above average sugar density, it was also selected due to a high salt content, which is a WHO Europe nutrient of concern [2].

To facilitate comparisons between countries and because energy density data were not available in the French data, the equivalent snack-based food groups were selected in the INCA2. However, because the French and UK foods did not match exactly (see Supplementary Material S2), analyses were undertaken in their separate datasets rather than merged into one; therefore differences between the two countries could not be statistically compared together. The main food groups analysed in the INCA2 were pâtisseries et gateaux (hereby referred to as ‘Cakes’), biscuits sucrés ou salés et barres (hereby referred to as ‘Biscuits and Crisps’) and chocolat (hereby referred to as ‘Chocolate’). Other studies have considered such foods energy-dense; Werle et al. [31] use cakes and crisps as examples of ‘highly caloric’ items and Wansink and Huckabee refer to the “the indulgent ‘C’ foods—cookies, cake, crackers, chips and candy” [32].

Within each of the selected main food groups homemade items were excluded in order to focus on commonly consumed, commercially available products, which have greater potential for FPS standardisation as part of potential future policy initiatives. This was done in the UK NDNS by searching for and excluding items with ‘homemade’ in the title on either the ‘SubFoodGroupDesc’ or ‘FoodName’ variable level. Homemade items were not always distinguished from those commercially available in the INCA2, therefore not all homemade items could be identified in the French data.

The remaining food items at the ‘FoodName’ level were then categorised based on product type, into newly created subgroups within each of the four main UK NDNS food groups selected. This was repeated for the three selected main food groups in the French INCA2 and food subgroups created to mirror those in the UK data where possible (see Supplementary Material S2). Miscellaneous items were categorised as ‘other’; for example ‘other’ cakes is made up of rum baba and blinis in the INCA2 and Chinese cakes and pastries, rice flour cakes and plain pastry in the NDNS. ‘Cookies’ included both ‘traditional’ cookies and ‘luxury’ American-style soft cookies, which tend to have larger FPS [10,21]. The energy density of each food subgroup was calculated to provide accompanying information which, coupled with estimated consumed FPS, could provide insights into the potential energy contributions of certain foods and, therefore, the setting of on-pack serving sizes.

### 2.3. Statistical Methods

In both the French and UK data the weighted mean consumed FPS and standard deviation (SD) per eating occasion for adults aged 19–64 years consuming each food subgroup were calculated and tabled. SDs more than half the mean FPS were used to indicate a wide spread of FPS within a food category. The food subgroup FPS per eating occasion was calculated within the French and UK datasets and defined as the total weight of food consumed in each subgroup divided by the consumption frequency per person. Each consumer contributed a single mean portion weight to the population mean for each food subgroup; this avoided means being skewed by non-consumers, who were excluded, or those who ate certain foods more frequently than others. The main food group FPS was calculated as the mean of all the mean food subgroup FPS. Analyses were restricted to adults aged 19–64 years to prevent distortion of results from children or the elderly, who may consume smaller portions; the underweight were also excluded from all analyses. Socio-demographic and dietary characteristics of those sampled from both surveys were determined and tabled. Normal weight was defined as having a BMI 18.5–25 kg/m^2^ and OWOB as ≥25 kg/m^2^. The difference in FPS reported by normal weight compared to OWOB adults aged 19–64 years consuming each food subgroup was t-tested in unadjusted analyses with BMI as a categorical variable. Two regression models were used, adjusting for sex and age and also under-reporting, age and sex to test for significant differences between FPS as the dependent variable and continuous BMI as an independent variable (predictor). To better utilise the detail provided in the datasets, continuous BMI was used in the adjusted regression analysis. STATA SE v14 and 15 [33,34] was used to analyse all data, which were weighted as explained above, and statistical significance set at *p* < 0.01 due to the large number of statistical tests performed.

Consumption frequency was defined as the total number of eating occasions of each food group per participant (including non-consumers), averaged to one day by dividing the consumption frequency per person by the number of completed diary days. An eating occasion was defined as any consumption incidence, regardless of FPS or number of units eaten. Analyses of consumption frequency was only possible in the UK data, as the INCA2 data had no means of identifying which individuals completed all seven diary days and, if not, how many days were completed; therefore, consumption frequency per person per day could not be calculated. Adjusted regression analysis was used to test for statistically significant associations between consumption frequency as the dependent and (continuous) BMI as the predictor after adjusting for age and sex (model 1) or adjusting for age, sex and excluding under-reporters (model 2).

A sensitivity analysis was conducted by repeating the analyses after identifying and excluding under-reporters. Under-reporters were included in the main analyses, following recommendations to minimise selection bias [35,36,37]. However, including under-reporters could potentially confound any relationship between FPS and BMI, as under-reporting is itself associated with higher BMI [7]. For this reason under-reporters were excluded as a sensitivity analysis and adjusted for in the main analyses. Under-reporter identification was based on energy intake of the whole diet rather than the specific food groups selected for review. Under-reporters were identified using participant height and weight data to generate basal metabolic rate (BMR) and BMR:energy intake ratio variables following the Oxford equations detailed in Henry [37]. These BMR and BMR:energy intake ratio variables were used to generate a low cut-off via the Goldberg method [38] using a physical activity level (PAL) of 1.55 across all individuals. 1.55 is an accepted value for a sedentary lifestyle in the populations used [39,40,41] and makes analysis of a large number of individuals more feasible because a common value is applied to all participants, and the large proportion with missing physical activity data can still be included. It also avoids the complexities and pitfalls of calculating individual PAL values; such as measures of moderate-vigorous activity being insufficiently accurate to estimate individual PAL values [41]. 

## 3. Results

There were 2117 adults aged 19–64 years in the French INCA2 and 3413 in the UK NDNS (excluding those with missing BMI or infeasible BMR:energy intake values). In the BMI analyses, 55% were normal weight and 40% OWOB (*n* = 1251; 866 respectively, *n* = unweighted) in the French and 37% normal weight and 61% OWOB (*n* = 1222; 2191 respectively) in the UK surveys (Table 1). In the INCA2 4% (*n* = 98) and in the NDNS 2% (*n* = 46) underweight adults aged 19–64 years were excluded from BMI analyses.

OWOB adults analysed in the INCA2 were significantly older, with a greater proportion of males, 32% higher weight, higher BMI and were less likely to have a degree compared to normal weight individuals. The profile of those analysed in the NDNS was similar; OWOB adults were older, had a greater proportion of males and were 34% heavier. However, French and UK nutritional profiles differed. French OWOB adults had a lower carbohydrate and total sugars consumption (41%Energy intake (%E) and 17%E respectively compared to 43%E and 18%E in normal weight adults), whereas UK OWOB adults only had a slightly lower total sugars consumption (19%E compared to 20%E in normal weight adults). In both countries under-reporters were more likely to be OWOB; in the INCA2 32% and in the NDNS 44% OWOB adults were estimated to be under-reporters of overall energy intake compared to 18% and 23%, respectively, of normal weight adults (Table 1).

### 3.1. Food Portion Size (FPS)

There was a large spread of FPS in certain food groups, particularly in France. All three selected French main food groups, but only Biscuits and Chocolate in the UK, had a SD greater than half the mean. In the INCA2 64% (*n* = 7) of Cakes subgroups had a SD greater than half of the mean FPS for all adults aged 19–64 years; for Biscuits and Crisps this was 100% (*n* = 12) and for Chocolate 89% (*n* = 8). In the UK NDNS 27% (*n* = 4) of Cakes subgroups had a SD greater than half of the mean FPS for all adults aged 19–64 years; for Biscuits this was 50% (*n* = 5); for Crisps this was 38% (*n* = 3) and for Chocolate 77% (*n* = 10).

In the French INCA2 the three food subgroups with the highest mean FPS for normal weight adults 19–64 years in Cakes were ‘fruit pie’ (181 g [SD 76 g]), ‘tart’ (170 g [60 g]) and ‘pastries’ (130 g [93 g]). For OWOB individuals this was ‘tart’ (182 g [SD 58 g]), ‘pastries’ (155 g [85 g]) and ‘fruit pie’ (154 g [44 g]). For Biscuits and Crisps in normal weight individuals this was ‘filled chocolate’ (61 g [40 g]), ‘unfilled coated biscuits with inclusions’ (52 g [51 g]) and ‘filled non-chocolate’ (47 g [32 g]) and for OWOB individuals this was ‘filled chocolate’ (60 g [39 g]), ‘unfilled coated biscuits with inclusions’ (43 g [29 g]) and both ‘cookies’ (42 g [23 g]) and ‘other’ (42 g [26 g]). For the Chocolate group in normal weight individuals this was ‘Mars-type bar’ (52 g [40 g]), ‘wafer bar’ (49 g [22 g]) and ‘chocolate spread’ (41 g [42 g]) and for OWOB individuals this was ‘wafer bar’ (46 g [19 g]), ‘Mars-type bar’ (38 g [15 g]) and ‘chocolate spread’ (31 g [25 g]) (see Table 2; for information on all adults see Appendix A).

In the UK NDNS the three food subgroups with the highest mean FPS for normal weight adults 19–64 years in Cakes were ‘pastries’ (93 g [30 g]), ‘fruit pie’ (91 g [38 g]) and ‘éclairs’ (89 g [46 g]) and for OWOB individuals this was ‘pastries’ (110 g [58 g]), ‘fruit pie’ (92 g [46 g]) and ‘doughnut’ (84 g [48 g]). For Biscuits in normal weight individuals this was ‘cookies and flapjack’ (47 g [37 g]), and ‘unfilled coated/inclusions’ (33 g [18 g]), ‘filled non-chocolate’ (33 g [24 g]) and ‘cereal bars’ (33 g [13 g]). For OWOB individuals this was ‘cookies and flapjack’ (43 g [30 g]), ‘jaffa cakes’ (42 g [49 g]) and ‘filled non-chocolate’ (35 g [19 g]). For Crisps in normal weight individuals this was ‘nuts’ (63 g [28 g]), ‘popcorn’ (50 g [31 g]) and ‘tortilla chips’ (44 g [39 g]) and for OWOB individuals this was ‘popcorn’ (123 g [98 g]), was ‘nuts’ (76 g [34 g]) and ‘tortilla chips’ (40 g [32 g]). For Chocolate in normal weight individuals this was ‘coated nuts/fruit’ (81 g [64 g]), ‘Mars-type bar’ (49 g [17 g]) and ‘other’ (48 g [28 g]) and for OWOB individuals this was ‘coated nuts/fruit’ (66 g [69 g]), ‘Mars-type bar’ (47 g [17 g]) and ‘honeycomb/crunch’ (44 g [37 g]) (see Table 3; for information on all adults see Appendix B). Food subgroups with the largest FPS were not necessarily the most energy dense (Appendix C); but there was also little variation in the energy density of the majority of the subgroups within each main food group, particularly in Chocolate.

There was little evidence of statistically significant FPS differences by BMI status (Table 2 and Table 3). However, on average the mean FPS of Cakes reported by French OWOB individuals was 44% larger than that reported by normal weight individuals—this was statistically significant. Little difference by BMI status was observed for the main UK Cake group. In contrast, French OWOB individuals reported smaller FPS than normal weight individuals in the majority of Biscuit, Crisps and Chocolate subgroups, although differences were not statistically significant. However, FPS of short biscuits reported by OWOB UK adults were significantly 30% larger than those reported by normal weight adults.

### 3.2. Adjusted Analyses of the Relationship between FPS and Body Mass Index (BMI)

When adjusting both for sex and age (model 1) and under-reporting, age and sex (model 2) in the regression analyses, there were just two French food subgroups with significant associations between FPS and BMI used as continuous variables, whereas there were none in the unadjusted analyses. Only total Cakes was statistically significant in both the unadjusted and the adjusted analyses and retained the same direction of association. Overall, after adjusting for sex and age (model 1), for every point increase in BMI between individuals, a difference of 3.1 g (99% CI 1.0 to 5.2 *p* =< 0.001) in their consumed FPS of ‘other cakes’ and 1.0 g (99% CI 0.1 to 1.9 *p* = 0.004) in ‘dark chocolate’ was observed in the same direction. In addition, the Cakes main food group FPS was significantly associated with BMI, where FPS increased by 1 g (99% CI 0.01 to 2.1 *p* = 0.01) with every BMI point increase. When adjusting for under-reporting, age and sex (model 2) FPS was positively associated with BMI in the same subgroups (see Supplementary Material S3 for all associations).

In the UK regression analysis (model 1), FPS was significantly associated with BMI in high-fat bar snacks only, which increased in FPS by 3.3 g (99% CI –0.1 to 6.7, *p* = 0.01) with every point increase in BMI after adjusting for sex and age. The same was true when adjusting for under-reporting, age and sex (model 2), where ‘high-fat bar snacks’ increased by 3.2 g (99% CI 0.2 to 6.3, *p* = 0.01) with every point increase in BMI. Unlike the French analyses, none of the main UK food groups were significantly associated with BMI (see Supplementary Material S4 for all associations).

### 3.3. Sensitivity Analysis Excluding Potential Under-Reporters

Of those who reported consuming Cakes in the French INCA2, 16% (*n* = 489 unweighted) under-reported overall energy intake and were excluded in the sensitivity analysis. In Biscuits and Crisps this was 12% (*n* = 247) and in Chocolate 15% (*n* = 211). In the adjusted analyses ‘other cakes’ lost significance after excluding under-reporters, likely due to lower number of individuals consuming foods in this subgroup and loss of power, but ‘dark chocolate’ remained significantly associated with higher FPS in OWOB individuals (data not shown). The Cakes main food group also retained and strengthened its association, with FPS increasing by a greater amount after excluding under-reporters.

In the UK NDNS, sensitivity analysis identified 23% (*n* = 635 unweighted) Cakes consumers as under-reporters of overall energy intake. In Biscuits consumers this was 31% (*n* = 1170), in Crisps 30% (*n* = 661) and in Chocolate 22% (*n* = 551) of consumers. ‘High fat bar snacks’ retained a higher FPS in OWOB individuals after excluding under-reporters, and like in the French INCA2, there was no change in the direction of association (data not shown).

### 3.4. Consumption Frequency

Analysis of consumption frequency as the dependent variable and BMI as the predictor was only possible in the UK data, as the INCA2 had no means of identifying how many diary days were completed for each individual from the available data, and therefore consumption frequency per day could not be calculated. When adjusting for age and sex (model 1) for every point increase in BMI, consumption frequency per day of Cakes and Chocolate decreased (Cakes: −0.006 99% CI −0.01 to −0.003 *p* =< 0.001; Chocolate −0.008 99% CI −0.01 to −0.004 *p* =< 0.001). When adjusting for age and sex after excluding under-reporters (model 2) Cakes was no longer significant, but for every point increase in BMI, consumption frequency per day still decreased in Chocolate −0.008 99% CI −0.01 to −0.001 *p* = 0.003) (Table 4). So although the higher an individual’s BMI, the less often these foods were consumed, in real terms there was negligible association.

## 4. Discussion

These analyses constitute a detailed examination of FPS and how these might vary with BMI in two large, nationally representative groups in energy, fat and sugar-dense snack foods. Of the main food groups analysed, only Cakes in the French INCA2 had a significant association between FPS and BMI, where FPS increased with each BMI point increase. There were very few significant associations between FPS and BMI in the energy-dense food subgroups analysed from the French and UK national dietary surveys, and these categories differed between the countries. The lack of UK and French similarities between the types of foods with significant associations suggests that French and UK diet preferences of OWOB individuals may differ across the selected main food groups. ‘Pastries’, ‘fruit pie’ and ‘Mars-type’ chocolate bars had the largest FPS for both normal and OWOB adults aged 19–64 years in both the French and UK analyses (Table 2 and Table 3). The consumed FPS information generated from these analyses is useful for our aim of adding to the body of knowledge that may help inform investigations into under-studied aspects of portion size, such as portion size policies and their acceptance by the public [20]. It could also help inform decisions around setting on-pack serving sizes, which have not been updated in the UK in over 20 years [22]. To set realistic on-pack serving sizes it would be helpful to know how much individuals consume in one sitting as a frame of reference, even if these do not necessarily become the on-pack serving size. 

On-pack serving sizes are not currently regulated or standardised and, unlike the requirement to state pack-size, providing serving-size information is not currently mandatory in the UK [42]. Without an on-pack serving-size, consumers may substitute pack-size as a unit of consumption, in a ‘unit bias’ that risks resulting in over-consumption and excess energy intake. This ‘unit bias’ could also result in individuals underestimating their consumed portion size, where they recognise that the whole unit is larger than an appropriate portion, but still eat the whole unit [43]. This is particularly relevant in snack foods, which are the focus of these analyses, and demonstrates the value of providing information that could help set on-pack serving sizes. Updated guidelines are, therefore, required, but should be realistic and formulated with sensitivity in order to avoid encouraging consumers to further increase consumed portion size. There is a fine balance to be struck in creating realistic, consistent on-pack serving-size guidance without encouraging consumers to eat larger portions or multiple units.

Mean FPS in the high FPS subgroups varied in their proximity to UK government FPS guidance, which was last updated in 1993 [19]. The three Cakes subgroups, ‘tortilla chips’ and ‘Mars-type bar’ were lower than the average suggested FPS in the guidance, whereas the Biscuits subgroups and remaining Chocolate and Crisps subgroups were higher [19]. However, with the exception of ‘Mars-type bar’, French mean FPS were higher than the UK guidance. This could be due to genuine differences in dietary patterns, or a function of under-reporting, which was higher in the UK. There was also a large spread of FPS within the food subgroups, particularly in the INCA2, which could be indicative of the lack of up-to-date FPS guidance in Europe [43,44] as there are no set guidelines for manufacturers or consumers to follow.

The few significant associations present were positive, showing that adults with higher BMIs reported consuming larger FPS. The extent to which this is a valid finding or one resulting from methodological limitations and misreporting cannot be fully determined. Studies have variously found few associations between FPS and adiposity [45], concluded that under-reporting masks associations between FPS and measures of adiposity [46] and found positive associations in certain energy-dense foods in adolescents after excluding misreporters [10]. Under-reporting masking associations could explain the few associations found in our adjusted analyses. In these associations, only small increases in FPS with each BMI point increase in the few food subgroups were seen; yet even small effect sizes could result in weight gain caused by increased energy intake over time.

Herman et al. [47] claim evidence is lacking to support excess energy intake via elevated FPS as a causal factor in the obesity epidemic, citing other factors, including consumption frequency, as potentially more significant. Mattes [48] found evidence that, in addition to FPS, consumption frequency also influences energy intake and, therefore, adiposity. Others have also found consumption frequency and energy intake to be positively associated, though this did not necessarily result in positive associations with BMI [49,50,51,52,53], potentially due to under-reporting. Conversely, in our adjusted analyses consumption frequency was negatively associated at negligible levels with BMI in Cakes and Chocolate, and in Chocolate after excluding under-reporters. Whilst unexpected, this highlights the difficulties presented by high under-reporting levels and other limitations with the cross-sectional data generated from NDS. Murakami and Livingstone [54] support this, citing differences in dietary assessment methodology, consumption frequency definitions, and approaches to under-reporting as explanations for the lack of consensus.

Under-reporting clearly presents a limitation in using NDS to assess FPS, affecting the number and variety of food main and subgroups with associations between FPS and BMI. Using the Goldberg method, under-reporting in the present analysis was estimated to be 1.5 times higher in the UK than in France, at 35% and 23% respectively. This is similar to levels previously reported, at 32% and 22.5% respectively [41,55]. This is possibly due to the greater proportion of OWOB individuals in the NDNS, with OWOB being associated with under-reporting in this analysis and elsewhere [36]. Compared to the overall under-reporting rates for the NDSs, there were lower percentages of under-reporters of energy intake who consumed the main energy-dense food categories analysed, indicating that under-reporters of energy intake are less likely to consume, or report consuming these snack type foods.

Vanrullen et al. [41] also found that snacking was associated with lower levels of under-reporting. This could be explained by social desirability bias; research suggests that foods perceived as less healthy, such as energy-dense snack foods, may be less likely to be reported [36,56]. Vanrullen et al. [41] also found that under-reporting was more attributable to consumption frequency than FPS. This suggests that including under-reporters would impact less on the accuracy of analyses because the under-reporting error would be concentrated more in individuals’ reporting of the consumption frequency rather than the FPS. This uncertainty in the extent and effect of under-reporting is problematic, as true consumption cannot be determined, creating challenges for effective policy formation across Europe.

### Strengths and Limitations

A limitation of our current study is the low number of consumers in some of the food subgroups, which may mean the analyses are underpowered to find associations at this level. An additional limitation is that it uses cross-sectional survey data, which cannot demonstrate the direction of association and, therefore, a causal relationship [35]. Nevertheless, our intention was not to imply that a single product could cause an individual to become OWOB, but to explore how reported FPS might vary with BMI. Longitudinal, prospective studies to track weight over time, with physical activity data to more accurately determine under-reporters, are part of the triangulation needed to understand the causal relationship between FPS and BMI. Most previous studies of FPS have focused on, or included short-term intake from a small number of participants in experimental studies, or used commercially available rather than average consumed FPS from large NDS samples [14,17,21,22,57,58].

In terms of strengths, the categorisation of foods by product type broadly matching the UK and French subgroups and examining FPS of similar products as they would be sold in a commercial environment and subsequently consumed by individuals, allows a more meaningful assessment of FPS than previous categorisations. Previous studies tend to use broader or incompatible food groups [23,44,45,46], where the diversity of foods in each group is higher and, therefore, comparability is lower. Future work could consider other energy-dense food and drinks such as fast food, breakfast cereals, ice cream, sugar-sweetened beverages or alcohol, as foods other than snack foods are related to BMI. Our focus on commercially available products provides information on FPS, split by BMI status, which could provide valuable evidence for policy development targeted at industry. Policies to reduce consumed FPS are needed now, and may have greater, more immediate impact across the population if targeted at popular, commercially available foods [15].

A further strength is that there are few studies exploring FPS across Europe, and the literature focuses primarily on America. However, as only two Western European countries were investigated, the ability to make Europe-wide assertions is limited; quality raw NDS data (as distinct from summary reports) with the necessary food code variables were not readily available to analyse from other countries. Nevertheless, the French and UK NDS used similar dietary methodologies of consecutive, self-reported food diaries (albeit with a different number of collection days) with estimated FPS, making them good comparative cases to report together.

Energy density and consumption frequency averaged to one day was not available for the French INCA2, so the main food groups selected for analysis were based on those chosen in the UK NDNS, and consumption frequency analysis limited to the UK. This made comparisons easier. Additionally, they are both developed Western European countries with some similarities in foods consumed and with similar dietary methodologies; however, different French and UK dietary patterns may result in differences in the most frequently consumed main food groups. This has implications for future extension of the analyses to other countries if their NDS lack consumption frequency data or methodologies differ. There is a need for better quality, harmonised NDS methodology and implementation across Europe, potentially achievable with new technologies, in order to generate robust data on which policy can be based. 

Further methodological limitations include the use of a 1.55 PAL across all individuals rather than being estimated individually, which limits the accuracy with which under-reporters can be identified, although 1.55 is an accepted value for a sedentary lifestyle in the populations used [39,40,41]. The French and UK data collection periods also differ, which limits the ability to make valid comparisons and highlights the need for regular, standardised data collection. In addition, French homemade items were not all demarcated, so could not be fully excluded, which may distort FPS and limit comparisons to the UK. This accentuates the possibility that the consumed FPS data do not necessarily represent commercially available industry-set FPS, thereby potentially losing some policy-making application to create realistic FPS guidance. Future work will explore how closely consumed FPS relate to pack-size and on-pack serving sizes in order to provide evidence to assist obesity-prevention policy decisions on reducing FPS and energy intake.

## 5. Conclusions

FPS for commonly consumed high-energy, -fat and -sugar foods were generated for two countries, which could provide important information for food policy work. There was limited evidence of associations between FPS and BMI of energy dense foods, and although the few significant associations found were positive, the subgroups with significant associations differed across the two countries. The limited evidence may be due to the cross-sectional nature and other limitations of the NDS data, such as the high estimates of under-reporting and the small number of individuals in some food subgroups. Excluding under-reporters impacted upon results; levels were higher in the UK, potentially due to the higher number of OWOB individuals previously associated with under-reporting. Future work should further explore data limitations like under-reporting, and investigate relationships between consumed FPS and on-pack serving sizes, which could help to inform future obesity-prevention policies.

## Figures and Tables

**Table 1:** General and dietary characteristics of all adults aged 19–64 y * in the French INCA2 and UK NDNS (Y1–6) dietary surveys.

**FRANCE INCA2**	**ALL (*n* = 2117)**			**NORMAL WEIGHT (*n* = 1251)**			**OWOB (*n* = 866)**			***p*-Value**
	**Mean**	**95% CI**		**Mean**	**95% CI**		**Mean**	**95% CI**		
**General characteristics**										
Age (year)	41	41	42	38	38	39	45	44	46	<0.001
Female (%)	50	47	53	55	51	58	43	39	47	<0.001
Height (cm) †	169	169	170	169	168	170	169	169	170	0.6
Weight (kg) †	72	71	73	63	63	64	83	82	84	<0.001
BMI (kg/m^2^) †	25	25	25	22	22	22	29	29	29	<0.001
Ethnicity (% white)	91	90	92	91	89	92	92	89	94	0.4
Education (% with degree)	17	15	19	21	18	24	11	9	14	<0.001
Under-reporters (%)	24	22	26	18	15	20	32	28	31	<0.001
**Dietary characteristics**										
Total energy (kcal)	2011	1978	2044	2001	1957	2044	2025	1975	2076	0.5
Fat (%E)	37	37	38	37	37	38	38	37	38	0.3
Carbohydrates (%E)	42	42	43	43	43	44	41	40	42	<0.001
Total sugars (%E)	18	17	18	18	18	19	17	16	17	<0.001
Salt (g)	6.9	6.7	7.0	6.7	6.5	6.9	7.0	6.8	7.3	0.02
Under-reporter energy intake (kcal)	1351	1315	1388	1256	1210	1302	1424	1371	1476	<0.001
**UK NDNS**	**ALL (*n* = 3413)**			**NORMAL WEIGHT (*n* = 1222)**			**OWOB (*n* = 2191)**			***p*-Value**
	**Mean**	**95% CI**		**Mean**	**95% CI**		**Mean**	**95% CI**		
**General characteristics**										
Age (year)	41	41	42	37	36	38	44	43	44	<0.001
Female (%)	50	48	52	57	53	61	46	43	49	<0.001
Height (cm) †	169	169	170	170	169	170	169	169	170	0.4
Weight (kg) †	79	78	79	65	64	66	87	86	88	<0.001
BMI (kg/m^2^) †	27	27	28	23	22	23	30	30	31	<0.001
Ethnicity (% white)	88	87	90	88	85	90	88	86	90	1.0
Education (% with degree)	28	26	30	34	31	38	25	22	27	0.02
Under-reporters (%)	36	34	38	23	20	26	44	41	47	<0.001
**Dietary characteristics**										
Total energy (kcal)	1861	1835	1887	1899	1852	1947	1838	1807	1869	0.03
Fat (%E)	33	33	33	33	33	34	33	32	33	0.004
Carbohydrates (%E)	46	46	46	46	45	47	46	45	46	0.7
Total sugars (%E)	19	19	20	20	19	20	19	19	19	0.002
Salt (g)	5.6	5.5	5.7	5.6	5.5	5.8	5.6	5.5	5.7	0.8
Under-reporter energy intake (kcal)	1368	1342	1394	1250	1207	1292	1404	1374	1435	<0.001

* Underweight adults with a BMI < 18.5 and adults with missing BMI or infeasible BMR: energy intake values were excluded from analyses. † Analyses exclude those adults with missing values.

**Table 2:** Mean portion size of energy-dense foods by BMI status for consumers aged 19–64 years in the French INCA2.

FOOD GROUP	NORMAL WEIGHT					OVERWEIGHT/OBESE					OWOB FPS as % of Normal Weight FPS
	*n* †	% Individuals Consuming **	Mean Food Portion Size (FPS) per Person (g)	99% CI		*n* †	% Individuals Consuming **	Mean FPS per Person (g)	99% CI		
**CAKES**											
**TOTAL ‡**	**1772**	**N/A**	**117**	**111**	**124**	**1120**	**N/A**	**133**	**125**	**141**	14 *
Other cakes and patisserie	17	1.0	83	60	105	15	0.6	119	70	168	44
Pancakes and brioche ‡	225	9.6	121	102	140	124	4.8	136	112	160	12
Chocolate cake and gateau	234	10.0	90	80	99	131	5.8	97	84	110	8
Cake and gateau non-chocolate ‡	285	13.5	102	90	114	173	7.2	119	103	136	17
Doughnut ‡	42	2.2	68	42	94	29	1.4	54	33	75	−21
Eclairs ‡	72	3.5	88	78	97	36	1.8	119	71	166	35
Fruit cake ‡	52	2.3	63	46	80	45	2.0	70	46	93	11
Fruit pie	19	0.7	181	120	242	7	0.3	154	109	200	−15
Muffins and mini cakes ‡	174	8.2	66	56	76	98	4.6	67	52	82	1
Pastries ‡	188	6.6	130	109	151	114	4.4	155	129	182	20
Tart	353	15.3	170	161	180	271	12.8	182	171	194	7
**BISCUITS & CRISPS**											
**TOTAL** **‡**	**1215**	**N/A**	**37**	**33**	**42**	**663**	**N/A**	**35**	**30**	**40**	**−5**
Other biscuits and crisps ‡	24	0.8	38	23	53	11	0.4	42	20	64	10
Unfilled uncoated biscuits ‡	128	5.8	40	32	47	59	2.7	34	23	45	−15
Cereal bars ‡	58	2.6	37	26	47	34	1.4	30	24	37	−17
Cookies ‡	40	1.6	44	31	56	24	1.6	42	24	60	−4
Savoury biscuits plain ‡	199	9.2	26	22	30	139	6.7	27	22	32	3
Filled chocolate biscuits ‡	109	4.8	61	50	71	59	2.8	60	43	77	−1
Filled non-chocolate biscuits ‡	44	1.8	47	33	61	19	1.0	36	19	53	−24
Potato crisps std. ‡	151	7.7	28	13	43	96	4.9	27	17	37	−5
Savoury biscuits flavoured ‡	53	2.2	37	25	50	28	0.8	33	22	44	−12
Short biscuits ‡	80	4.0	38	21	55	48	2.0	31	19	43	−19
Tortilla chips‡	13	0.8	24	−6	55	6	0.3	14	2	27	−41
Unfilled coated biscuits with inclusions ‡	94	4.4	52	34	70	34	1.4	43	29	58	−17
**CHOCOLATE**											
**TOTAL** **‡**	**1009**	**N/A**	**27**	**24**	**31**	**517**	**N/A**	**25**	**22**	**29**	**−7**
Chocolate spread ‡	183	8.8	41	28	55	72	3.3	31	22	46	−24
Chocolate with additions ‡	117	5.5	31	21	41	60	2.6	30	22	37	−5
Dark chocolate ‡	163	7.0	12	10	15	94	4.3	17	12	22	40
Honeycomb/crunch ‡	23	1.0	25	11	39	5	0.1	27	−9	63	8
Mars type bar ‡	65	3.0	52	37	67	22	1.0	38	29	47	−27
Milk chocolate ‡	304	13.0	19	16	22	176	7.4	22	16	27	16
Truffles ‡	103	3.5	23	14	31	59	2.0	30	13	46	32
Wafer bar	39	1.7	49	38	59	23	1.1	46	35	56	−6
White chocolate ‡	12	0.7	37	3	70	6	0.3	18	6	30	−50

* *p* =< 0.01. † Unweighted number of individuals consuming each food subgroup. The total *n* value represents all Cake/Biscuit and Crisp/Chocolate types consumed by the sample, so may be larger than the number of individuals in the sample (as detailed in Table 1), as some individuals will consume multiple subgroups. ** Percentages will not total 100%, as the weighted percentage is of all adults aged 19–64 rather than just those particular food groups. ‡ standard deviation (SD) greater than half the mean FPS per person for all adults aged 19–64 years.

**Table 3:** Mean portion size of energy-dense foods by BMI status for consumers aged 19–64 years in the UK NDNS (Y1-6).

FOOD GROUP	NORMAL WEIGHT					OVERWEIGHT/OBESE					OWOB FPS as % of Normal Weight FPS
	*n* †	% Individuals Consuming **	Mean FPS per Person (g)	99% CI		*n* †	% Individuals Consuming **	Mean FPS per Person (g)	99% CI		
**CAKES**											
**TOTAL**	**1028**	**N/A**	**69**	**64**	**75**	**1555**	**N/A**	**68**	**64**	**73**	**−1**
Other ‡	31	1.2	42	21	64	41	1.2	66	49	83	56
Teacakes	69	2.2	62	52	71	115	3.1	64	56	71	4
Cake and gateau non-choc ‡	82	2.1	56	36	74	146	3.9	49	42	55	−12
Swiss roll ‡	35	1.0	49	28	69	70	1.6	61	37	86	26
Doughnut	61	2.0	71	61	82	80	2.6	84	60	109	18
Croissant	84	2.8	72	57	87	82	2.3	70	57	84	−2
Muffins and cupcakes	75	2.3	67	55	79	108	3.0	70	57	83	4
Chocolate cake and gateau	79	2.6	76	50	102	75	2.0	67	56	77	−12
Bars and slices	51	1.1	46	34	59	79	2.1	39	33	46	−15
Fruit pie	66	2.0	91	74	108	110	2.5	92	78	107	2
Éclairs	22	0.6	89	54	124	38	1.3	68	56	81	−23
Tart	40	1.1	61	46	76	67	2.0	75	62	89	24
Scones, pancakes and sweet dough ‡	94	1.8	73	49	97	154	2.6	67	53	80	−9
Pastries	44	1.5	93	79	107	69	2.1	110	84	137	19
Fruit cake and malt loaf	30	0.9	64	53	76	57	1.5	70	54	85	8
**BISCUITS**											
**TOTAL** **‡**	**1368**	**N/A**	**32**	**29**	**34**	**2236**	**N/A**	**33**	**31**	**34**	**3**
Unfilled coated/inclusions	239	6.2	33	29	37	402	9.5	33	30	37	2
Unfilled uncoated ‡	214	5.7	29	21	37	433	9.9	27	24	30	−6
Filled non-chocolate	153	4.3	33	27	38	245	6.2	35	31	38	6
Cereal bars	118	3.1	33	30	37	175	4.3	33	30	35	−3
Cookies and flapjack ‡	130	3.7	47	34	60	167	4.5	43	36	50	−9
Short biscuits ‡	108	3.0	21	17	24	178	4.5	27	22	32	30 *
Savoury biscuits plain ‡	246	7.2	27	24	30	378	9.2	28	25	32	6
Savoury biscuits flavoured	56	2.0	24	19	29	69	2.2	24	19	30	0
Jaffa cakes ‡	53	1.4	32	27	36	77	1.4	42	25	58	30
Filled chocolate	44	1.2	32	25	39	87	2.2	31	27	35	−3
**CRISPS**											
**TOTAL**	**741**	**N/A**	**32**	**29**	**35**	**1301**	**N/A**	**30**	**29**	**32**	**−6**
Potato and vegetable crisps std.	422	12.2	31	28	35	756	19.7	30	28	31	−6
Corn/maize snack	78	2.1	23	18	28	119	3.2	26	21	30	11
Potato snack shapes and puffed	96	2.9	24	21	28	201	5.8	26	23	29	7
Tortilla chips ‡	62	2.0	44	30	58	77	2.1	40	28	52	−9
Potato crisps crinkle	45	1.4	39	32	46	88	2.0	38	33	42	−3
Popcorn ‡	9	0.3	50	27	72	12	0.3	123	12	233	147
High-fat bar snacks ‡	22	0.6	21	13	30	39	1.4	33	20	46	53
Nuts	4	0.2	63	11	114	5	0.2	76	20	133	22
**CHOCOLATE**											
**TOTAL** **‡**	**994**	**N/A**	**37**	**34**	**40**	**1321**	**N/A**	**39**	**36**	**43**	**5**
Other ‡	15	0.3	48	27	69	32	0.8	33	23	43	−32
Milk chocolate ‡	271	7.9	33	28	38	287	7.3	38	30	46	14
Mars type bar	185	5.7	49	44	53	288	7.3	47	44	50	−3
Wafer bar	109	2.8	29	25	33	179	5.3	33	29	36	13
Caramel ‡	78	2.2	33	25	41	108	2.5	37	27	47	12
Sugar coated ‡	37	1.1	41	13	69	32	0.8	28	17	38	−33
Dark chocolate ‡	58	1.7	25	11	40	60	1.9	32	7	57	24
Honeycomb/crunch	49	1.7	35	26	43	75	1.6	44	30	58	25
Crème filled ‡	52	1.1	38	25	51	99	2.1	36	28	44	−6
Truffles ‡	41	1.2	26	19	33	47	1.3	26	13	39	1
White chocolate ‡	22	0.7	29	9	49	22	0.6	28	−1	56	−5
Chocolate with additions ‡	46	1.6	37	25	49	68	2.0	45	35	56	22
Coated nuts/fruit ‡	31	0.9	81	43	119	24	0.9	66	20	112	−18

* *p* =< 0.01. † Unweighted number of individuals consuming each food subgroup. The total *n* value represents all Cake/Biscuit/Crisp/Chocolate types consumed by the sample, so may be larger than the number of individuals in the sample (as detailed in Table 1), as some individuals will consume multiple subgroups. ** Percentages will not total 100%, as the weighted percentage is of all adults aged 19–64 rather than just those particular food groups. ‡ SD greater than half the mean FPS per person for all adults aged 19–64 years.

**Table 4:** Association between consumption frequency (CF) of energy dense food groups in adults aged 19–64 years and BMI in the NDNS. Model 1 adjusted for age and sex. Model 2 adjusted for age and sex after excluding under-reporters.

MAIN FOOD GROUP	MODEL 1 (*n* = 3413)				MODEL 2 (*n* = 2142)			
	Difference in CF *	99% CI		*p*-Value	Difference in CF *	99% CI		*p*-Value
**Cakes**	−0.006	−0.01	−0.003	<0.001	−0.004	−0.01	0.001	0.05
**Biscuits**	−0.005	−0.01	0.0009	0.03	0.005	−0.004	0.01	0.2
**Crisps**	0.0003	−0.003	0.004	0.8	0.004	−0.002	0.009	0.07
**Chocolate**	−0.008	−0.01	−0.004	<0.001	−0.008	−0.01	−0.001	0.003

* Difference in consumption frequency (number of times eaten per day) with each point increase in BMI.

**Figure A1:**
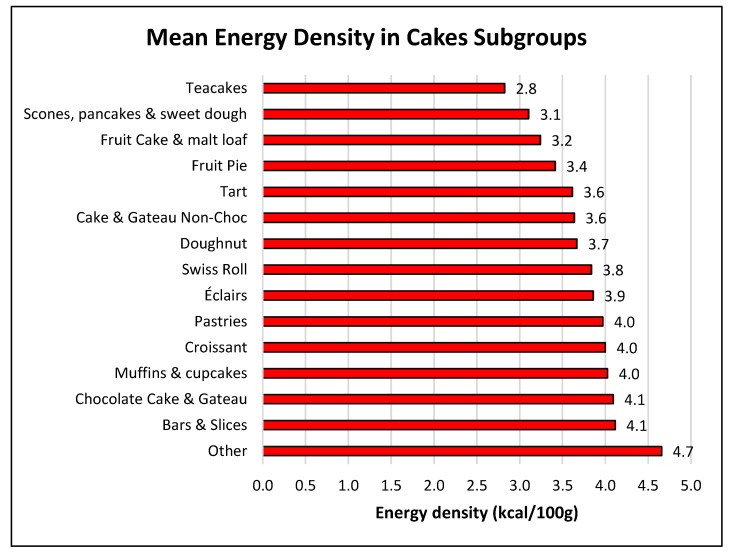

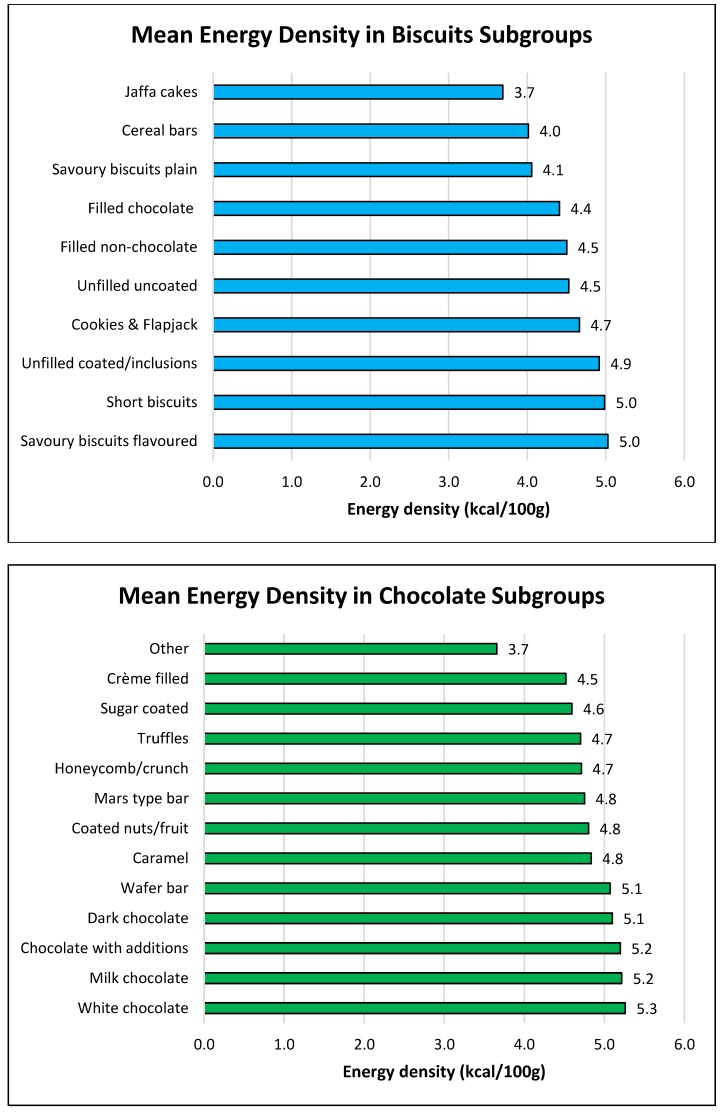

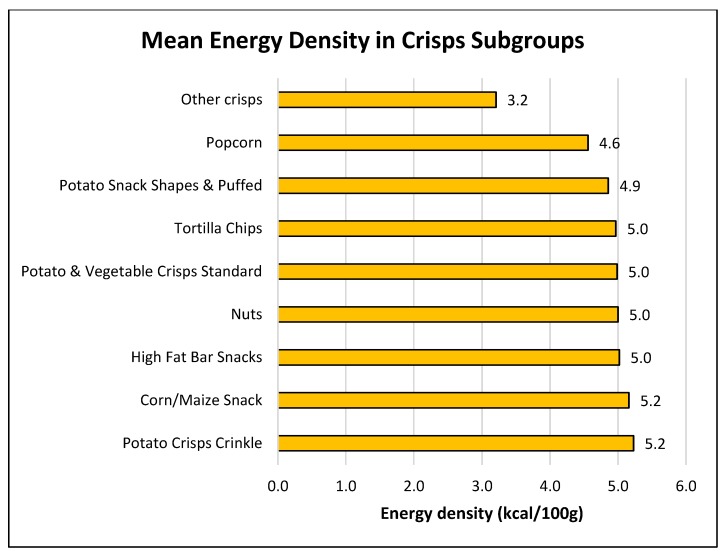
Mean energy density in food subgroups in each of the selected main food groups in the UK NDNS.

## Supplementary Materials

The following are available online at http://www.mdpi.com/2072-6643/11/1/12/s1, Table S1: Combining data from Years 1–4 and Years 5 and 6, Table S2: Food subgroups in the selected main food groups in the French INCA2* and UK NDNS, Table S3: Associations between portion size of energy-dense foods and BMI for adults aged 19–64 y in the French INCA2. Model 1 adjusted for sex and age. Model 2 adjusted for under-reporting, sex and age, Table S4: Associations between portion size of energy-dense foods and BMI for adults aged 19–64 y in the UK NDNS (Y1–6). Model 1 adjusted for sex and age. Model 2 adjusted for under-reporting, sex and age.

## Appendix A

**Table A1:** Mean portion size of energy-dense foods for all consumers aged 19–64 years in the French INCA2.

FOOD GROUP	*n* †	% Individuals Consuming (Weighted) **	Mean FPS per Person (g)	SD	99% CI	
**CAKES**						
**TOTAL** **‡**	**3078**	**N/A**	**123**	**66**	**118**	**128**
Other cakes and patisserie	33	1.7	96	45	70	122
Pancakes and brioche ‡	368	15.2	128	101	113	144
Chocolate cake and gateau	397	17.0	91	45	84	98
Cake and gateau non-chocolate ‡	481	21.8	108	69	98	117
Doughnut ‡	77	3.7	64	52	46	81
Eclairs ‡	116	5.6	98	52	80	115
Fruit cake ‡	105	4.6	65	41	51	78
Fruit pie	29	1.1	169	73	126	212
Muffins and mini cakes ‡	302	14.2	66	43	58	73
Pastries ‡	318	11.8	139	90	123	154
Tart	657	29.5	175	60	168	183
**BISCUITS & CRISPS**						
**TOTAL** **‡**	**2020**	**N/A**	**36**	**31**	**33**	**39**
Other biscuits and crisps ‡	42	1.5	41	28	28	55
Unfilled uncoated biscuits ‡	201	9.0	38	29	32	44
Cereal bars ‡	97	4.2	34	24	27	41
Cookies ‡	72	3.5	42	29	31	52
Savoury biscuits plain ‡	353	16.8	26	21	23	29
Filled chocolate biscuits ‡	177	7.9	61	40	52	70
Filled non-chocolate biscuits ‡	71	3.1	42	29	32	52
Potato crisps std. ‡	267	13.5	28	39	18	37
Savoury biscuits flavoured ‡	86	3.2	36	24	27	46
Short biscuits ‡	142	6.5	36	40	25	47
Tortilla chips ‡	24	1.4	24	27	5	43
Unfilled coated biscuits with inclusions ‡	142	6.4	49	46	36	62
**CHOCOLATE**						
**TOTAL** **‡**	**1632**	**N/A**	**27**	**25**	**24**	**29**
Chocolate spread ‡	272	12.9	38	38	29	48
Chocolate with additions ‡	194	8.8	31	30	24	37
Dark chocolate ‡	274	12.0	14	13	12	16
Honeycomb/crunch ‡	32	1.3	26	24	14	38
Mars type bar ‡	92	4.2	47	36	36	58
Milk chocolate ‡	514	21.6	20	22	17	23
Truffles ‡	167	5.8	25	33	17	33
Wafer bar	67	3.0	47	21	40	54
White chocolate ‡	20	1.0	32	30	7	56

† Unweighted number of individuals consuming each food subgroup. ** Percentages will not total 100%, as the weighted percentage is of all adults aged 19–64 rather than just those particular food groups. ‡ SD greater than half the mean FPS per person for all adults aged 19–64 years.

## Appendix B

**Table A2:** Mean portion size of energy-dense foods for all consumers aged 19–64 years in the UK NDNS (Y1–6).

FOOD GROUP	*n* †	% Individuals Consuming (Weighted) **	Mean FPS per Person (g)	SD	99% CI	
**CAKES**						
**TOTAL**	**2739**	**N/A**	**69**	**28**	**66**	**72**
Other ‡	78	2.5	53	29	39	67
Teacakes	197	5.6	63	23	58	69
Cake and gateau non-choc ‡	241	6.3	51	26	43	58
Swiss roll ‡	110	2.8	55	34	40	70
Doughnut	151	4.9	78	29	64	92
Croissant	177	5.6	71	30	62	81
Muffins and cupcakes	194	5.7	69	23	60	77
Chocolate cake and gateau	161	4.8	71	30	56	86
Bars and slices	138	3.5	42	17	36	48
Fruit pie	186	4.8	93	35	83	103
Éclairs	62	1.9	77	32	61	93
Tart	112	3.3	73	33	62	84
Scones, pancakes and sweet dough ‡	263	4.8	68	35	56	80
Pastries	117	3.7	104	41	87	120
Fruit cake and malt loaf	95	2.6	68	28	58	78
**BISCUITS**						
**TOTAL** **‡**	**3823**	**N/A**	**33**	**17**	**31**	**34**
Unfilled coated/inclusions	678	16.7	33	16	31	36
Unfilled uncoated ‡	685	16.4	28	20	25	31
Filled non-chocolate	425	11.4	34	15	31	37
Cereal bars	316	8.0	33	8	31	35
Cookies and flapjack ‡	312	8.8	45	24	38	52
Short biscuits ‡	301	7.9	25	14	22	29
Savoury biscuits plain ‡	657	17.4	28	14	26	30
Savoury biscuits flavoured	134	4.4	24	10	21	28
Jaffa cakes ‡	139	3.0	37	24	29	45
Filled chocolate	139	3.6	32	10	28	36
**CRISPS**						
**TOTAL**	**2178**	**N/A**	**31**	**14**	**30**	**33**
Potato and vegetable crisps std.	1260	34.1	30	12	29	32
Corn/maize snack	215	5.9	25	10	22	29
Potato snack shapes and puffed	306	9.1	26	8	23	28
Tortilla chips ‡	146	4.3	42	25	34	51
Potato crisps crinkle	145	3.6	38	10	35	42
Popcorn ‡	25	0.7	85	53	31	140
High-fat bar snacks ‡	64	2.1	30	20	21	39
Nuts	9	0.4	70	27	36	105
**CHOCOLATE**						
**TOTAL** **‡**	**2457**	**N/A**	**39**	**21**	**36**	**41**
Other ‡	50	1.2	38	28	47	45
Milk chocolate ‡	590	16.1	35	26	31	40
Mars type bar	502	13.7	48	13	45	50
Wafer bar	306	8.5	31	10	29	34
Caramel ‡	197	4.9	35	18	29	41
Sugar coated ‡	71	2.0	36	20	19	53
Dark chocolate ‡	127	4.0	30	29	16	44
Honeycomb/crunch	133	3.6	40	19	32	48
Crème filled ‡	162	3.4	37	24	30	43
Truffles ‡	93	2.7	26	18	19	32
White chocolate ‡	47	1.3	30	18	14	46
Chocolate with additions ‡	121	3.8	44	26	35	53
Coated nuts/fruit ‡	58	1.9	73	56	44	102

† Unweighted number of individuals consuming each food subgroup. ** Percentages will not total 100%, as the weighted percentage is of all adults aged 19–64 rather than just those particular food groups. ‡ SD greater than half the mean FPS per person for all adults aged 19–64 y.
