# Supplementary material for: Portion Size of Energy-Dense Foods among French and UK Adults by BMI Status
Source: Nutrients. 2018 Dec 20;11(1):12. doi: 10.3390/nu11010012 (PMC6356251; doi:10.3390/nu11010012)
Supplement: Supplementary file 1 [file nutrients-11-00012-s001.zip › HR_Supplementary4_Nutrients.docx]

**Supplementary Material 4.** Associations between portion size of energy-dense foods and BMI for adults aged 19-64y in the UK NDNS (Y1-6). *Model 1 adjusted for sex and age. Model 2 adjusted for under-reporting, sex and age*.

| **FOOD GROUP** | **MODEL 1** | | | | **MODEL 2** | | | |
| --- | --- | --- | --- | --- | --- | --- | --- | --- |
|  | **Change in FPS (g) with each BMI point increase** | **99% CI** | | **Adjusted p-value** | **Change in FPS (g) with each BMI point increase** | **99% CI** | | **Adjusted p-value** |
| **Cakes** | | | | |  |  |  |  |
| **TOTAL** | **-0.1** | **-0.7** | **0.4** | **0.5** | **0.1** | **-0.5** | **0.7** | **0.6** |
| Other | 2.0 | -0.3 | 4.2 | 0.03 | 2.0 | -0.2 | 4.3 | 0.02 |
| Teacakes | 0.2 | -0.9 | 1.3 | 0.6 | 0.4 | -0.8 | 1.6 | 0.4 |
| Cake & Gateau Non-Choc | -0.4 | -1.6 | 0.8 | 0.4 | -0.6 | -2.4 | 1.2 | 0.4 |
| Swiss Roll | 0.5 | -1.8 | 2.9 | 0.6 | 0.9 | -1.5 | 3.3 | 0.3 |
| Doughnut | 2.1 | -1.6 | 5.8 | 0.1 | 1.9 | -0.8 | 4.6 | 0.07 |
| Croissant | 0.4 | -1.6 | 2.4 | 0.6 | 0.5 | -1.6 | 2.6 | 0.5 |
| Muffins & cupcakes | -0.2 | -1.7 | 1.3 | 0.8 | -0.1 | -1.6 | 1.4 | 0.9 |
| Chocolate Cake & Gateau | -0.7 | -2.7 | 1.3 | 0.3 | -0.5 | -2.5 | 1.4 | 0.5 |
| Bars & Slices | -0.8 | -1.8 | 0.2 | 0.04 | -0.5 | -1.4 | 0.4 | 0.2 |
| Fruit Pie | 0.7 | -1.2 | 2.6 | 0.3 | 1.1 | -0.8 | 3.1 | 0.1 |
| Éclairs | 1.0 | -0.9 | 2.9 | 0.2 | 1.3 | -0.5 | 3.2 | 0.07 |
| Tart | 0.6 | -1.2 | 2.5 | 0.4 | 0.9 | -1.2 | 3.1 | 0.3 |
| Scones, pancakes & sweet dough | -0.7 | -2.3 | 0.9 | 0.3 | -0.8 | -2.8 | 1.3 | 0.3 |
| Pastries | 0.7 | -1.3 | 2.8 | 0.4 | 1.8 | -1.4 | 5.0 | 0.1 |
| Fruit Cake & malt loaf | 1.7 | -0.3 | 3.7 | 0.03 | 1.7 | -0.3 | 3.7 | 0.03 |
| **Biscuits** | | | | |  |  |  |  |
| **TOTAL** | **0.1** | **-0.1** | **0.4** | **0.3** | **0.2** | **-0.1** | **0.5** | **0.07** |
| Unfilled coated/inclusions | 0.2 | -0.3 | 0.6 | 0.3 | 0.2 | -0.2 | 0.7 | 0.2 |
| Unfilled uncoated | -0.01 | -0.4 | 0.4 | 1.0 | 0.02 | -0.4 | 0.4 | 0.9 |
| Filled non-chocolate | 0.4 | -0.1 | 0.9 | 0.05 | 0.4 | -0.1 | 0.9 | 0.06 |
| Cereal bars | -0.3 | -0.7 | 0.1 | 0.1 | -0.3 | -0.7 | 0.1 | 0.09 |
| Cookies & Flapjack | 0.1 | -1.0 | 1.1 | 0.9 | 0.2 | -0.9 | 1.3 | 0.7 |
| Short biscuits | 0.3 | -0.2 | 0.7 | 0.1 | 0.3 | -0.1 | 0.8 | 0.07 |
| Savoury biscuits plain | 0.02 | -0.3 | 0.4 | 0.9 | 0.2 | -0.2 | 0.5 | 0.2 |
| Savoury biscuits flavoured | 0.1 | -0.6 | 0.7 | 0.8 | -0.01 | -0.6 | 0.5 | 1.0 |
| Jaffa cakes | 0.2 | -1.1 | 1.4 | 0.7 | 0.3 | -1.0 | 1.7 | 0.6 |
| Filled chocolate | 0.1 | -0.6 | 0.9 | 0.6 | 0.3 | -0.4 | 1.0 | 0.3 |
| **Crisps** | | | | |  |  |  |  |
| **TOTAL** | **-0.03** | **-0.4** | **0.3** | **0.8** | **0.05** | **-0.3** | **0.4** | **0.7** |
| Potato & Vegetable Crisps Std | -0.1 | -0.4 | 0.2 | 0.4 | -0.1 | -0.4 | 0.2 | 0.6 |
| Corn/Maize Snack | 0.9 | -0.4 | 2.3 | 0.07 | 1.0 | -0.4 | 2.3 | 0.06 |
| Potato Snack Shapes & Puffed | 0.2 | -0.3 | 0.8 | 0.3 | 0.3 | -0.4 | 0.9 | 0.3 |
| Tortilla Chips | -0.9 | -2.7 | 0.9 | 0.2 | -0.5 | -2.2 | 1.3 | 0.5 |
| Potato Crisps Crinkle | -0.1 | -1.0 | 0.7 | 0.6 | -0.1 | -0.9 | 0.7 | 0.7 |
| Popcorn | 4.1 | -1.0 | 9.2 | 0.04 | 4.1 | -1.2 | 9.5 | 0.05 |
| High Fat Bar Snacks | 3.3 | -0.1 | 6.7 | 0.01 | 3.2 | 0.2 | 6.3 | 0.01 |
| Nuts | -6.3 | -20.4 | 7.9 | 0.3 | 4.6 | -9.7 | 18.8 | 0.4 |
| **Chocolate** | | | | |  |  |  |  |
| **TOTAL** | **0.1** | **-0.4** | **0.5** | **0.6** | **0.1** | **-0.4** | **0.6** | **0.6** |
| Other | -1.1 | -4.0 | 1.7 | 0.3 | -0.9 | -3.4 | 1.9 | 0.4 |
| Milk chocolate | -0.2 | -0.8 | 0.5 | 0.5 | -0.2 | -1.0 | 0.5 | 0.4 |
| Mars type bar | -0.1 | -0.6 | 0.4 | 0.6 | -0.2 | -0.7 | 0.4 | 0.4 |
| Wafer bar | 0.1 | -0.4 | 0.6 | 0.7 | 0.1 | -0.5 | 0.6 | 0.8 |
| Caramel | 0.4 | -0.7 | 1.5 | 0.4 | 0.3 | -0.8 | 1.4 | 0.5 |
| Sugar coated | -2.1 | -5.4 | 1.2 | 0.1 | -2.0 | -5.2 | 1.2 | 0.1 |
| Dark chocolate | 1.8 | -2.9 | 6.4 | 0.3 | 2.1 | -2.6 | 6.9 | 0.3 |
| Honeycomb/crunch | 0.7 | -0.7 | 2.2 | 0.2 | -0.2 | -1.3 | 0.9 | 0.6 |
| Crème filled | -0.2 | -1.8 | 1.3 | 0.7 | -0.1 | -1.7 | 1.5 | 0.9 |
| Truffles | -0.1 | -0.9 | 0.8 | 0.8 | 0.02 | -0.9 | 0.9 | 1.0 |
| White chocolate | -0.4 | -2.7 | 1.9 | 0.6 | -0.4 | -2.7 | 1.9 | 0.6 |
| Chocolate with additions | 0.2 | -1.0 | 1.4 | 0.6 | 0.4 | -0.9 | 1.8 | 0.4 |
| Coated nuts/fruit | -1.8 | -7.8 | 4.2 | 0.4 | -1.6 | -7.4 | 4.1 | 0.5 |
