# Supplementary material for: Portion Size of Energy-Dense Foods among French and UK Adults by BMI Status
Source: Nutrients. 2018 Dec 20;11(1):12. doi: 10.3390/nu11010012 (PMC6356251; doi:10.3390/nu11010012)
Supplement: Supplementary file 1 [file nutrients-11-00012-s001.zip › HR_Supplementary1_Nutrients.docx]

**Combining data from Years 1-4 and Years 5&6**

The NDNS datasets for Year 1-4 and Year 5&6 can be combined for analysis of Years 1-6 but in order to produce valid results the two sets of weights need to be re-scaled. This will ensure that the two sets of data are in the correct proportion i.e. 2:1. A different calculation is required for each weight (individual, nurse, blood etc).

Re-scaling is necessary because there were more participants *per year* in years 1-4 than in years 5&6. In total, there are 6,828 participants in the Year 1-4 data but only 2,546 participants in Year 5&6 data. The ratio of these totals is (approx.) 2.68:1. Therefore, if the weights were combined into one variable without any further adjustment, years 1-4 would have more weight *per year* than years 5&6.

To re-scale the weights correctly, it is necessary to perform the following calculations:

1. Divide each weight variable by its sum (i.e. the sum of the weights);
2. Multiply both by the total (combined) sum of the two weights;
3. Multiply the Year 1-4 weight by 2/3 and the Year 5&6 weight by 1/3.

You can then combine the resulting weights into one variable.

**Example: individual weights**

For example, to create new individual weights for analysis of the combined dataset, the steps would be as follows:

1. Divide Year 1-4 weight by 6828 and the Year 5&6 weight by 2546;
2. Multiply both weights by (6828 + 2546);
3. Multiply the Year 1-4 weight by 2/3 and the Year 5&6 weight by 1/3;
4. Combine the resulting weights into one variable.

To do all of this in in SPSS you could use the following syntax:

compute WTI_UKY1234r = WTI_UKY1234 * (6828 + 2546) / 6828 * (2/3).

compute WTI_UKY56r = WTI_UKY56 * (6828 + 2546) / 2546 * (1/3).

compute WTI_UKY1to6 = sum (WTI_UKY1234r , WTI_UKY56r).

Running these commands will result in a new set of weights (WTI_UKY1to6 ) for analysing Years 1-6. They should have a mean of 1 and be non-missing for all 9,374 cases in the combined dataset. You can check this by running descriptives on the weights:

desc WTI_UKY1to6.

Notes:

1. The intermediate weights (WTI_UKY1234r and WTI_UKY56r) can be discarded/deleted.

2. In this example the sum of the weights is equal to the total sample size in each case. This will not hold for subgroups (see below).

**Combining other weights**

Analogous calculations can be performed for:

Nurse weights (WTN_UKY1234 and WTN_UKY56)

Blood weights (WTB_UKY1234 and WTB_UKY56)

RPAQ weights (WTR_UKY1234 and WTR_UKY56)

24-hour urine weights (WTU_UKY1234 and WTU_UKY5) {* see note below}

* Note that 24-hour urine samples were only collected in Years 1-5 so when combining these weights, the correct proportions to use are 4/5 and 1/5 (rather than 2/3 and 1/3).

**Weights for combined sub-group analysis**

The above explanation assumes that analysis will be performed for all cases i.e. all adults and children. If analysis of subgroups is required then analogous calculations should be performed on the combined dataset filtered to include only the subgroup of interest. This will produce bespoke weights for analysis of that particular subgroup (adults only for example).

One additional step is required but otherwise the procedure is the same:

1. Divide each weight variable by its sum (i.e. the sum of the weights);
2. Multiply both by the total (combined) sum of the two weights;
3. Multiply the Year 1-4 weight by 2/3 (4/5 for the 24 hour urine weights) and the Year 5&6 weight by 1/3 (1/5 for the 24 hour urine weights);
4. Combine the resulting weights into one variable;
5. Re-scale this weight to have a mean of 1.

The additional step (v) ensures that the resulting weights have a mean of 1.

**Example: individual weights (adults only)**

To create new individual weights for analysis of adults only in the combined dataset, you could use the following syntax in SPSS:

select if age>=19.

desc WTI_UKY1234 WTI_UKY56 / stat = sum. {*}

compute WTI_UKY1234r = WTI_UKY1234 * (5391.3596 + 2012.2877) / 5391.3596 * (2/3).

compute WTI_UKY56r = WTI_UKY56 * (5391.3596 + 2012.2877) / 2012.2877 * (1/3).

compute WTI_UKY1to6 = sum (WTI_UKY1234r , WTI_UKY56r).

desc WTI_UKY1to6. {*}

compute WTI_UKY1to6 = WTI_UKY1to6 / 1.562610.

* these lines are optional **but required to produce the figures in the following commands**.

The resulting weights (WTI_UKY1to6 ) should have a mean of 1 and be non-missing for all 4,738 cases in the combined dataset of adults. As above, you can check this by running descriptives on the weights:

desc WTI_UKY1to6.

Notes:

1. Again, the intermediate weights WTI_UKY1234r & WTI_UKY56r can be discarded/deleted.
2. If subgroup analysis is performed using weights produced for the whole dataset, years 1-4 and 5&6 may not be in the correct proportion.
