# Supplementary material for: Portion Size of Energy-Dense Foods among French and UK Adults by BMI Status
Source: Nutrients. 2018 Dec 20;11(1):12. doi: 10.3390/nu11010012 (PMC6356251; doi:10.3390/nu11010012)
Supplement: Supplementary file 1 [file nutrients-11-00012-s001.zip › HR_Supplementary2_Nutrients.docx]

#### Supplementary material 2: Food subgroups in the selected main food groups in the French INCA2* and UK NDNS

| **FRANCE INCA2** | **UK NDNS** |
| --- | --- |
| **Pâtisseries et Gateaux** | **Buns, Cakes, Pastries & Fruit Pies** |
| Pancakes and Brioche | Scones, pancakes & sweet dough |
| Chocolate Cake & Gateau | Chocolate Cake & Gateau |
| Cake & Gateau Non-Chocolate | Cake & Gateau Non-Chocolate |
| Doughnut | Doughnut |
| Eclairs | Éclairs |
| Fruit cake | Fruit Cake & malt loaf |
| Fruit Pie | Fruit Pie |
| Muffins and Mini Cakes | Muffins & cupcakes |
| Pastries | Pastries |
| Tart | Tart |
|  | Teacakes |
|  | Swiss Roll |
|  | Bars & Slices |
|  | Croissant |
| Other cakes and patisserie | Other cakes |
| **Biscuits Sucrés ou Salés et Barres** | **Biscuits** |
| Unfilled uncoated biscuits | Unfilled uncoated biscuits |
| Cereal bars | Cereal bars |
| Cookies | Cookies & Flapjack |
| Savoury biscuits plain | Savoury biscuits plain |
| Filled chocolate biscuits | Filled chocolate biscuits |
| Filled non-chocolate biscuits | Filled non-chocolate biscuits |
| Savoury biscuits flavoured | Savoury biscuits flavoured |
| Short biscuits | Short biscuits |
| Unfilled coated biscuits with inclusions | Unfilled coated biscuits with inclusions |
| Potato crisps std | Jaffa cakes |
| Tortilla chips |  |
| Other biscuits and crisps | Other biscuits |
| **Chocolat** | **Chocolate Confectionary** |
| Chocolate spread | Other |
| Milk chocolate | Milk chocolate |
| Mars type bar | Mars type bar |
| Wafer bar | Wafer bar |
| Dark chocolate | Dark chocolate |
| Honeycomb/crunch | Honeycomb/crunch |
| Truffles | Truffles |
| White chocolate | White chocolate |
| Chocolate with additions | Chocolate with additions |
|  | Caramel |
| * The French INCA2 data lacks the variables required to calculate energy density of particular food groups; therefore subgroups were created to match as closely as possible those created from the NDNS. | Sugar coated |
|  | Crème filled |
|  | Coated nuts/fruit |
|  | **Crisps & Savoury Snacks** |
|  | Potato & Vegetable Crisps Standard |
|  | Corn/Maize Snack |
|  | Potato Snack Shapes & Puffed |
|  | Tortilla Chips |
|  | Potato Crisps Crinkle |
|  | Popcorn |
|  | High Fat Bar Snacks |
|  | Nuts |
