# Supplementary material for: Portion Size of Energy-Dense Foods among French and UK Adults by BMI Status
Source: Nutrients. 2018 Dec 20;11(1):12. doi: 10.3390/nu11010012 (PMC6356251; doi:10.3390/nu11010012)
Supplement: Supplementary file 1 [file nutrients-11-00012-s001.zip › HR_Supplementary3_Nutrients.docx]

**Supplementary Material 3.** Associations between portion size of energy-dense foods and BMI for adults aged 19-64y in the French INCA2. *Model 1 adjusted for sex and age. Model 2 adjusted for under-reporting, sex and age*.

| **FOOD GROUP** | **MODEL 1** | | | | **MODEL 2** | | | |
| --- | --- | --- | --- | --- | --- | --- | --- | --- |
|  | **Change in FPS (g) with each BMI point increase** | **99% CI** | | **Adjusted p-value** | **Change in FPS (g) with each BMI point increase** | **99% CI** | | **Adjusted p-value** |
| **Cakes** | | | | |  |  |  |  |
| **TOTAL** | **1.0** | **0.01** | **2.1** | **0.01** | **1.2** | **0.1** | **2.3** | **0.003** |
| Other cakes and patisserie | 3.1 | 1.0 | 5.2 | <0.001 | 4.4 | 1.5 | 7.4 | <0.001 |
| Pancakes and Brioche | 1.3 | -3.0 | 5.5 | 0.4 | 1.5 | -2.8 | 5.7 | 0.4 |
| Chocolate Cake & Gateau | 1.3 | -0.4 | 2.9 | 0.05 | 1.3 | 0.3 | 3.0 | 0.04 |
| Cake & Gateau Non-Chocolate | 1.2 | -1.2 | 3.5 | 0.2 | 1.5 | -0.7 | 3.8 | 0.08 |
| Doughnut | -0.4 | -5.4 | 4.6 | 0.9 | -0.1 | -5.0 | 4.9 | 1.0 |
| Eclairs | 2.6 | -1.1 | 6.3 | 0.1 | 3.0 | -1.1 | 7.2 | 0.06 |
| Fruit cake | 1.7 | -1.5 | 4.8 | 0.2 | 1.6 | -1.1 | 4.4 | 0.1 |
| Fruit Pie | -2.4 | -14.0 | 9.1 | 0.6 | -3.2 | -15.0 | 8.6 | 0.5 |
| Muffins and Mini Cakes | 0.04 | -2.0 | 2.1 | 1.0 | 0.1 | -1.8 | 2.0 | 0.9 |
| Pastries | 0.3 | -2.9 | 3.4 | 0.8 | 0.3 | -2.8 | 3.5 | 0.8 |
| Tart | 1.0 | -1.0 | 3.1 | 0.2 | 1.3 | -0.9 | 3.4 | 0.1 |
| **Biscuits & Crisps** | | | | |  |  |  |  |
| **TOTAL** | **0.1** | **-0.4** | **0.5** | **0.7** | **0.1** | **-0.5** | **0.6** | **0.8** |
| Other biscuits and crisps | -0.3 | -4.0 | 3.4 | 0.8 | -0.8 | -4.4 | 2.8 | 0.6 |
| Unfilled uncoated | 0.1 | -1.1 | 1.3 | 0.9 | 0.2 | -1.1 | 1.4 | 0.8 |
| Cereal bars | 0.03 | -0.6 | 0.7 | 0.9 | 0.1 | -0.7 | 0.9 | 0.8 |
| Cookies | 0.5 | -1.7 | 2.7 | 0.6 | 0.4 | -1.9 | 2.7 | 0.6 |
| Savoury biscuits plain | 0.4 | -0.3 | 1.0 | 0.1 | 0.5 | -0.2 | 1.1 | 0.05 |
| Filled chocolate | -0.05 | -2.4 | 2.3 | 1.0 | 0.2 | -2.1 | 2.5 | 0.8 |
| Filled non-chocolate | -0.2 | -1.6 | 1.1 | 0.6 | -0.2 | -1.7 | 1.3 | 0.7 |
| Potato crisps std. | 0.1 | -0.7 | 0.9 | 0.8 | -0.4 | -1.5 | 0.7 | 0.3 |
| Savoury biscuits flavoured | -0.2 | -2.0 | 1.5 | 0.7 | -0.6 | -2.5 | 1.2 | 0.4 |
| Short biscuits | -0.9 | -3.2 | 1.3 | 0.3 | -0.6 | -3.0 | 1.7 | 0.5 |
| Tortilla chips | -0.4 | -2.7 | 2.0 | 0.7 | -0.3 | -2.7 | 2.1 | 0.8 |
| Unfilled coated and/or inclusions | 0.1 | -2.4 | 2.6 | 0.9 | 0.1 | -2.5 | 2.6 | 1.0 |
| **Chocolate** | | | | |  |  |  |  |
| **TOTAL** | **0.3** | **-0.4** | **0.9** | **0.3** | **0.3** | **-0.3** | **0.9** | **0.2** |
| Chocolate spread | -0.8 | -3.1 | 1.5 | 0.4 | -0.6 | -2.8 | 1.6 | 0.5 |
| Chocolate with additions | 0.3 | -1.3 | 2.0 | 0.6 | 0.3 | -1.4 | 2.0 | 0.7 |
| Dark chocolate | 1.0 | 0.1 | 1.9 | 0.004 | 0.9 | 0.2 | 1.7 | 0.001 |
| Honeycomb/crunch | -1.7 | -4.5 | 1.0 | 0.1 | -1.8 | -4.7 | 1.1 | 0.1 |
| Mars type bar | -1.6 | -4.0 | 0.9 | 0.1 | -1.6 | -4.0 | 0.9 | 0.1 |
| Milk chocolate | 0.4 | -0.6 | 1.3 | 0.3 | 0.3 | -0.6 | 1.3 | 0.4 |
| Truffles | 1.7 | -1.5 | 4.9 | 0.2 | 1.3 | -0.9 | 3.4 | 0.1 |
| Wafer bar | -0.4 | -1.8 | 1.0 | 0.5 | -0.6 | -1.9 | 0.8 | 0.3 |
| White chocolate | -1.7 | -6.9 | 3.6 | 0.4 | 2.6 | -2.5 | 7.8 | 0.2 |
